# *Citrobacter freundii* induces sepsis with new-onset status seizure in an adult: A case report and literature review

**DOI:** 10.1097/MD.0000000000032549

**Published:** 2023-01-06

**Authors:** Xiao-bo Chen, Ya-xiong Zhou, Yan Feng

**Affiliations:** a Department of General Surgery, Shuangliu District First People’s Hospital, Chengdu, Sichuan, China; b Emergency Department, West China Hospital, Sichuan University, Chengdu, People’s Republic of China; c Department of Anesthesiology, West China Hospital, Sichuan University & The Research Units of West China (2018RU12), Chinese Academy of Medical Sciences, Chengdu, Sichuan, China.

**Keywords:** brain abscess, *Citrobacter freundii*, sepsis

## Abstract

Sepsis with brain abscess induced by Citrobacter freundii in adults has rarely been reported and is extremely pathogenic with a high mortality rate. Early diagnosis of the infection still remains challenging.

**Patient Concerns:**

A 27-year-old woman was transferred to our emergency department for fever, status seizure and refractory hypotension. Administration of midazolam, propofol and sodium valproate could not attenuate the seizure except for the addition of vecuronium. The blood white blood cell count was 42.91 (109/L) with 80% neutrophils, and procalcitonin was 22.46ng/mL.

**Diagnoses:**

Both deoxyribonucleic acid and ribonucleic acid of C freundii were detected in blood by metagenomic next-generation sequencing of pathogens,the diagnosis of septic shock and brain abscess caused by C freundii was highly suspected.

**Interventions:**

On day 1, antibiotics of cefoperazone sodium and sulbactam sodium 6g/day, immunoglobulin, and hydrocortisone were used with suspected septic shock. Continuous renal replacement therapy was used to eliminate excessive lactate, ammonia, myohemoglobin and creatinine. On day 2, a brain computed tomography scan revealed multiple patchy slightly low densities in the brain, antibiotics were adjusted to meropenem intravenously 3g/day.

**Outcomes:**

On day 2, a brain computed tomography scan revealed multiple patchy slightly low densities in the brain, she died on day 3.

**Lessons:**

Clinicians should consider the possibility of brain abscess when evaluating a patient with new-onset dizziness, fever, seizure, or other neurologic symptoms or signs, especially for patients whose mental status changes. metagenomic next generation sequencing and resistance genes could be considered when cerebrospinal fluid or blood results are negative and clinical manifestations are highly suspected of infection or when the treatment time is limited.

## 1. Introduction

Among the 13 *Citrobacter* species, *Citrobacter freundii* and *Citrobacter koseri* have been most frequently associated with human disease.^[1]^ *C freundii* induces a broad spectrum of infections as an opportunistic pathogen, including infections of the urinary tract, respiratory tract, wounds, and bloodstream that occurs in immunocompromised persons.^[2]^ Bloodstream infection of *Citrobacter* in adults is less common^[3]^ and sepsis induced by *C freundii* in adults has rarely been reported. We report a female adult who developed sepsis with new onset of status seizure and diagnosed with *C freundii* meningitis who died from system failure and septic shock. The unique feature of meningitis caused by *Citrobacter species* is their frequent association with brain abscess formation.^[4]^ Since the disease is extremely pathogenic with a high mortality rate, early diagnosis and treatment are crucial for survival. This article provides some interesting viewpoint, which may be valuble for the future clinical diagnosis and management of *C freundii* brain abscess.

## 2. Case presentation

**Figure 1.**
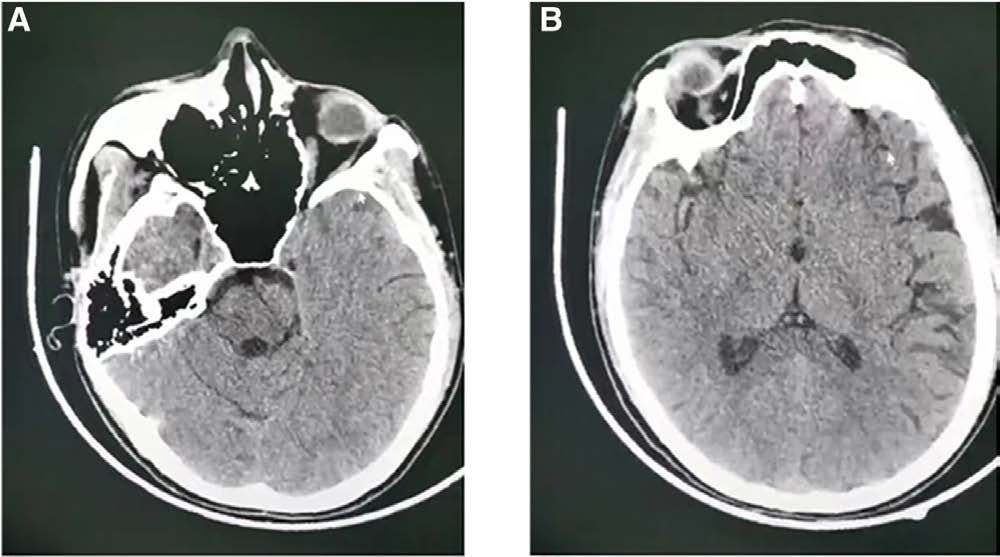
Brain computed tomography imaging of the patient at onset. (A, B) No definite hemorrhagic foci and large infarcts in brain parenchyma were found.

**Figure 2.**
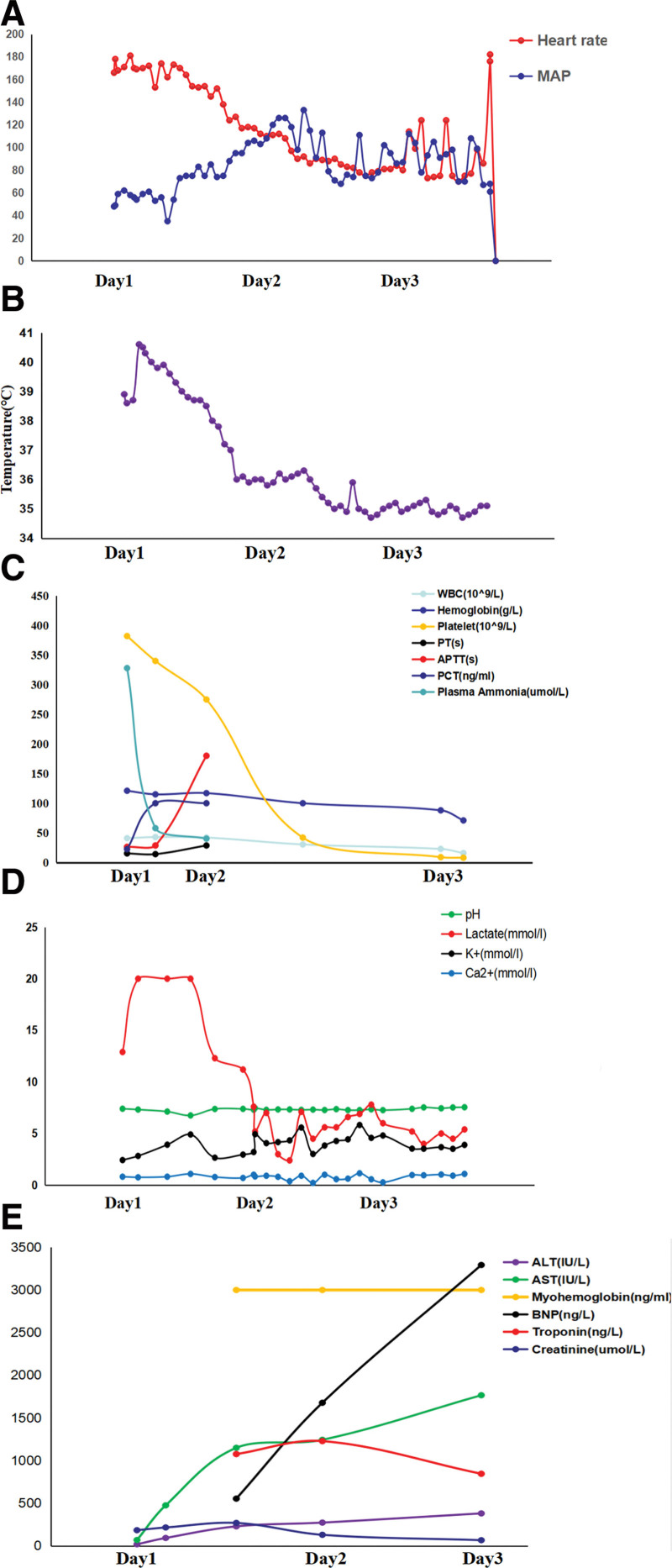
Clinical course of heart rate and mean arterial pressure (A), temperature (B). Clinical course of laboratory data: blood white blood cell, hemoglobin, platelet, prothrombin time (PT), activated partial thromboplastin time (APTT), procalcitonin (PCT), and plasma ammonium (C); pH, lactate, potassium ion (K^+^), calcium ion (Ca^2+^) (D); ALT, AST, myohemoglobin, BNP, troponin, creatinine (E). ALT = alanine aminotransferase, AST = aspertate aminotransferase, BNP = brain natriuretic peptide, PCT = procalcitonin.

A 27-years-old woman was transferred to our emergency department for fever, status seizure and refractory hypotension. Some clinical manifestations including insomnia, dizziness, occasional headache, eating less have been reported and piperacillin, tazobactam, desphenidol and moxifloxacin were taken in recent twelve days. Six hours before admission, she experienced a sudden onset of dizziness, headache, and vomiting and then rapidly developed seizure and incontinence without a known history of febrile seizures or epilepsy. She was evaluated at a nearby hospital, and a computed tomography (CT) scan of the brain showed no definite hemorrhagic foci or large infarcts in the brain parenchyma (Fig. 1), then intubated for hypoxia and transferred to our hospital. Physical examination revealed consciousness disorder (Glasgow coma scale 2; E1V1VT), a temperature of 38.9°C, a blood pressure of 73/36 mm Hg, a pulse rate of 175 bpm, and an oxygen saturation of 100% (mechanical ventilation mode: SIMV (A/C + PC): f = 16 breaths/minutes, PI: 11 cm H_2_O, FiO_2_: 45%, PEEP: 5 cm H_2_O). Administration of midazolam, propofol and sodium valproate could not attenuate the seizure except for the addition of vecuronium. Norepinephrine was supplied continuously to maintain stable circulation. Therapeutic hypothermia (32–34°C) was applied. The blood white blood cell count was 42.91 (109/L) with 80% neutrophils, and procalcitonin was 22.46 ng/ml. Her clinical course is shown in Figure 2. The Sequential Organ Failure Assessment score was 14. Antibiotics of cefoperazone sodium and sulbactam sodium 6 g/day, immunoglobulin, and hydrocortisone were used with suspected septic shock. Toxicology screening was negative. Continuous renal replacement therapy was used to eliminate excessive lactate (>20 mmol/L), ammonia (327 µmol/L), myohemoglobin (>3000 ng/mL) and creatinine (218 µmol/L).

**Figure 3.**
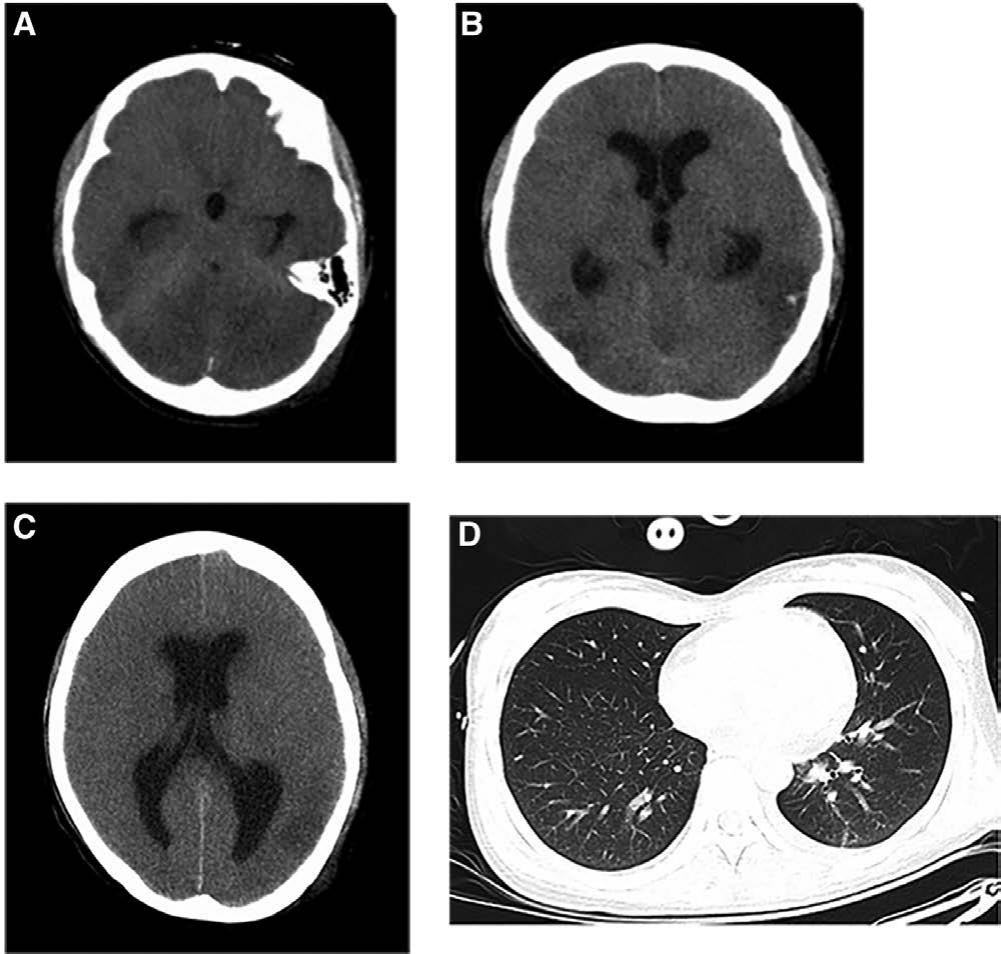
Brain and chest computed tomography imaging of the patient on day 2. Low density in bilateral cerebellar hemisphere, compression of the fourth ventricle, and interstitial cerebral edema (A), bilateral temporal occipital lobe and interstitial cerebral edema (B), lateral ventricular hydrocephalus (C); Chest computed tomography showed slight inflammation in both lower lungs (D).

**Figure 4.**
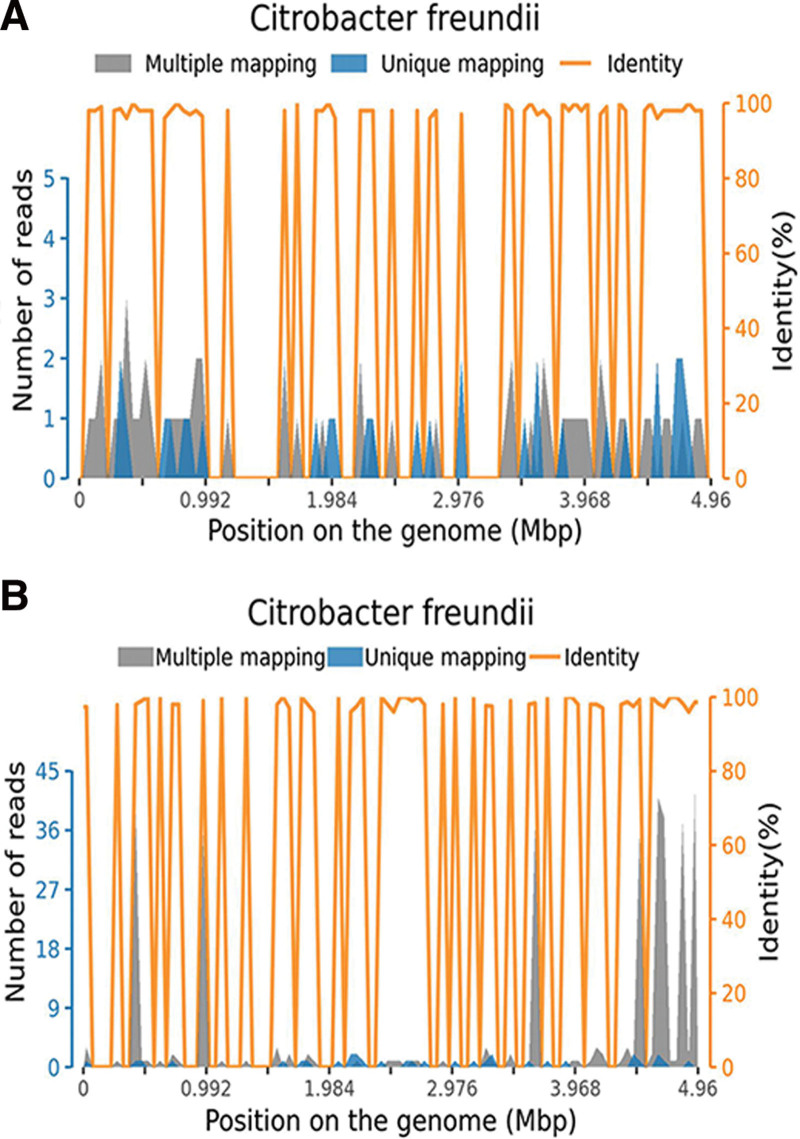
DNA and RNA of the *Citrobacter freundii* were detected in blood by metagenomic next-generation sequencing of pathogens, DNA = deoxyribonucleic acid, RNA = ribonucleic acid.

On day 2, a brain CT scan revealed multiple patchy slightly low densities in the bilateral cerebellar hemisphere, bilateral temporal occipital lobe, compression of the fourth ventricle, lateral ventricular hydrocephalus, and interstitial cerebral edema. Chest CT scan showed slight inflammation in both lower lungs (Fig. 3). Although the blood and cerebrospinal fluid (CSF) cultures were negative, antibiotics were adjusted to meropenem intravenously 3 g/day. Transfusion of plasma and platelets to address coagulation dysfunction. She died on day 3, and both deoxyribonucleic acid and ribonucleic acid of *C freundii* were detected in blood by metagenomic next-generation sequencing (mNGS) (Genskey, China) of pathogens (Fig. 4). Hence, the diagnosis of *C freundii* brain abscess was taken into account.

## 3. Discussion

At present, 31 cases of sepsis caused by *C freundii* from 1979 to 2021 have been reported.^[5–11]^ Among 4 *C freundii* sepsis adult patients, 1 was diagnosed with meningitis and eventually died. *C freundii* sepsis with meningitis was rarely reported, the mortality and morbidity rates are unacceptably high,^[12]^ The pathogenesis of meningitis and brain abscess may be related to the invasion of endothelial cells and result in the restriction of macroelements passing through the blood–brain barrier in the case of immunodeficiency.^[13]^

In our case, her history of insomnia and eating less can lead to impared immunity. Symptoms of sudden onset of dizziness, headache, and vomiting and then rapidly developed sepsis, seizure and incontinence without a known history of febrile seizures or epilepsy, administration of midazolam, propofol and sodium valproate could not attenuate the seizure except for the addition of vecuronium and negative blood and CSF cultures, which increased the difficulty in diagnosis and treatment of the disease. Nevertheless, neurological signs, fever, new onset of seizures, mental status changes, ranging from lethargy to coma were associated with meningitis or brain abscess.^[14]^ Additionally, CT scan revealed multiple patchy slightly low densities in the brain and interstitial cerebral edema were similar to those of other brain abscesses: equal- or low-density mass with low-density perifocal edema by CT scan.^[15,16]^ Negative results in the CSF and blood may be associated with prolonged use of broad-spectrum antibiotics and the formation of an abscess capsule without invading the meninges.^[15]^ mNGS evidence supported the diagnosis of *C freundii* sepsis with brain abscess.

Treatment of brain abscesses caused by pathogens, including surgery to remove the lesion and the use of appropriate antibiotics based on drug susceptibility testing.^[15]^ In our case, the patient deteriorated rapidly, surgery was not performed for unstable circulation and coagulation dysfunction. Bacteria of *C freundii* has been demonstratedly sensitive to cefoperazone and carbapenem.^[17]^ However, the pathogen had poor sensibility to theses antibiotic in our case. Possible reasons are as follows: First, the abscess was located in the brain parenchyma and did not invade the brain stroma or meninges, which made the antibiotics can not reach the lesion; Second, because of its insidious onset (dizziness, occasional headache), the diagnosis of this disease was delayed rendering treatment difficult; Third, drug-resistant *C freundii* strains may be present in this patient, multidrug-resistant *C freundii* strains are common and have been associated with a higher rate of in-hospital mortality than susceptible strains.^[18]^ Unfortunately, we did not detect resistance genes in *C freundii*. It has been suggested that the combination of ciprofloxacin and meropenem is the most appropriate therapy for *Citrobacter* brain infection because of good penetration into the CNS and neutrophils, low toxicity, and favorable sensitivity data.^[19]^ Currently, carbapenems, fourth-generation cephalosporins, amikacin, and quinolones are still reliable agents for drug-resistant *C freundii* strains.^[20]^

In summary, clinicians should consider the possibility of brain abscess when evaluating a patient with new-onset dizziness, fever, seizure, or other neurologic symptoms or signs. mNGS and resistance genes could be considered when CSF and blood cultures are negative or clinical manifestations are highly suspected of infection or when the treatment time is limited. Performing needle biopsy or lesion resection and appropriate antibiotic therapies in the early stage are essential for decreasing *C freundii* bacteria mortality as well as drug-resistant strains.

## Acknowledgements

The authors would like to thank the supporting of the funding.

## Author contributions

**Data curation:** Xiao-bo Chen, Ya-xiong Zhou.

**Investigation:** Ya-xiong Zhou, Yan Feng.

**Methodology:** Xiao-bo Chen, Yan Feng.

**Supervision:** Yan Feng.

**Visualization:** Xiao-bo Chen, Yan Feng.

**Writing – original draft:** Xiao-bo Chen.

**Writing – review & editing:** Ya-xiong Zhou, Yan Feng.
